# Clinical effectiveness of high-flow nasal cannula oxygen therapy in post-cardiac surgery hypoxemia: A retrospective cohort study

**DOI:** 10.1097/MD.0000000000047735

**Published:** 2026-02-20

**Authors:** Kaifeng Luo, Gang Li, Chengli Wang

**Affiliations:** a Department of Critical Care Medicine, 3201 Hospital of Xi’an Jiaotong University Health Science Center, Hanzhong, China.

**Keywords:** cardiac surgery, critical care, high-flow nasal cannula oxygen therapy, hypoxemia, respiratory failure

## Abstract

This retrospective cohort study aims to evaluate the clinical effectiveness of high-flow nasal cannula oxygen therapy (HFNCO) in managing hypoxemia following cardiac surgery. Baseline characteristics were comparable between groups (*P* > .05). At 6 hours post-extubation, the HFNCO group demonstrated significantly lower respiratory rate (20.16 vs 22.19 breaths/min, *P* = .001), higher pH (7.42 vs 7.46, *P* = .001), reduced PaCO_2_ (36.66 vs 40.35 mm Hg, *P* = .001), and improved P/F ratio (298.05 vs 237.45, *P* < .001). High-flow nasal cannula oxygen therapy reduced intensive care unit length of stay (ICU LOS; 3.21 vs 7.32 days, *P* < .001), total hospital LOS (23.69 vs 33.07 days, *P* = .002), reintubation rate (2% vs 14%, *P* = .002), and ICU delirium incidence (5% vs 17%, *P* = .007). No mortality difference was observed (*P* > .05). High-flow nasal cannula oxygen therapy was associated with improved oxygenation, enhanced CO_2_ clearance, and a reduction in clinical complications in post-cardiac surgery hypoxemia. It may be a valuable alternative to conventional oxygen therapy, warranting further validation in multicenter trials. A total of 203 patients with hypoxemia (PaO_2_ < 60 mm Hg, SpO_2_ < 90%, or P/F ≤ 300 mm Hg) after cardiac surgery (January 2021–January 2024) were included. Participants were divided into conventional oxygen therapy (n = 101) and HFNCO (n = 102) groups. Primary outcomes included respiratory rate, blood gas parameters (pH, PaCO_2_, PaO_2_/FiO_2_), and secondary outcomes encompassed ICU/LOS, reintubation rate, and ICU-acquired delirium. Statistical analyses were performed using SPSS 26.0.

## 1. Introduction

The incidence of cardiovascular diseases in China has been increasing annually, with some patients requiring cardiac surgery for therapeutic intervention. However, postoperative complications often prolong intensive care unit (ICU) stays and adversely affect prognosis, even posing life-threatening risks.^[1]^ Pulmonary dysfunction, particularly hypoxemia and acute respiratory distress syndrome, occurs in 3.5% to 11.7% of post-cardiac surgery cases.^[2]^ Postoperative cardiac instability and shallow breathing due to pain further exacerbate hypoxemia, necessitating respiratory support to improve outcomes. Traditional oxygen therapies, such as masks or nasal cannulas, have limitations. High-flow nasal cannula oxygenation (HFNCO), delivering heated and humidified oxygen at 8 to 80 L/min with adjustable FiO_2_ (21–100%), has gained clinical traction.^[3]^ Studies indicate HFNCO enhances oxygenation, reduces reintubation rates, shortens respiratory support duration,^[4,5]^ and improves mucociliary clearance by minimizing airway resistance and dead space ventilation.^[6]^ Despite its widespread ICU use, HFNCO’s effectiveness in post-cardiac surgery patients remains debated.^[5]^ This study aims to compare HFNCO with conventional oxygen therapy (COT) in managing hypoxemia after cardiac surgery to provide more robust evidence for its application in this specific population.

## 2. Materials and methods

### 2.1. Materials

This retrospective study included 203 patients with hypoxemia (PaO_2_ < 60 mm Hg, SpO_2_ < 90%, PaO_2_/FiO_2_ ≤ 300 mm Hg) after cardiac surgery (e.g., valve replacement, coronary artery bypass grafting) at our hospital from January 2021 to January 2024. Patients were divided into COT (n = 101) and HFNCO (n = 102) groups based on post-extubation oxygen therapy. Treatment allocation was determined by physician preference and device availability during the study period, following institutional oxygenation protocols. This study was approved by the Ethics Committee of 3201 Hospital Affiliated to Xi’an Jiaotong University (Approval No. LL-KYLW-2024-037). The requirement for informed consent was waived due to the retrospective nature of the study.

#### 2.1.1. Inclusion criteria

Age ≥ 18 years; mechanical ventilation ≥ 24 hours; passed spontaneous breathing trial; good compliance.

#### 2.1.2. Exclusion criteria

Contraindications to HFNCO; chronic neuromuscular or severe organ failure; chronic lung diseases (e.g., chronic obstructive pulmonary disease).

### 2.2. Interventions

#### 2.2.1. COT group

Received oxygen via nasal cannula (1–5 L/min) or mask (5–10 L/min), maintaining SpO_2_ at 90% to 99%.

#### 2.2.2. HFNCO group

Received heated (37°C) and humidified oxygen via high-flow nasal cannula (Fisher & Paykel Healthcare, Guangzhou, China) with initial settings: FiO_2_ 50% to 80%, flow rate 30 to 60 L/min, adjusted based on tolerance and effectiveness.

### 2.3. Outcome measures

A retrospective analysis was performed using electronic medical records. After extubation, patients received either COT or HFNCO per standardized institutional protocols. Data collected encompassed baseline characteristics; vital signs and arterial blood gases at 0.5 hours pre-extubation and 6, 12, and 24 hours post-extubation; and clinical outcomes (reintubation, length of stay, delirium, and mortality) until discharge or death. All data were anonymized and analyzed using a standardized database.

Vital signs (HR, RR, MAP) were continuously monitored and recorded from bedside monitors. Arterial blood gas samples were drawn from indwelling radial artery catheters and analyzed immediately using a blood gas analyzer. The oxygenation index (P/F ratio) was calculated as PaO_2_/FiO_2_. ICU delirium was assessed twice daily by trained ICU nurses using the Confusion Assessment Method for the ICU.

### 2.4. Statistical analysis

Data were analyzed using IBM SPSS Statistics for Windows, Version 26.0 (IBM Corp., Armonk). Continuous variables are expressed as mean (95% CI) or median (Q1, Q3). Categorical variables are presented as frequencies. Independent *t* tests, Mann–Whitney *U* tests, or χ^2^ tests were applied as appropriate. *P* < .05 indicated statistical significance. Baseline covariates including APACHE-II scores, mechanical ventilation duration, and surgical types were included in sensitivity analyses, confirming outcome robustness.

## 3. Results

### 3.1. Baseline characteristics

This study enrolled 203 participants, with 101 assigned to the COT group and 102 to the HFNCO group. Statistical analysis of baseline characteristics, including age, sex, smoking history, comorbidities, operative duration, mechanical ventilation duration, APACHE-II score, and pre-extubation parameters (HR, RR, arterial pH, MAP, SpO_2_, PaO_2_, PaCO_2_, and P/F ratio), revealed no significant differences between the 2 groups (*P* > .05). Detailed data are presented in Table 1.

**Table 1:** **Comparison of baseline characteristics between conventional oxygen therapy and high-flow nasal cannula oxygenation groups O(E)/M (Q1, Q3**).

Variable	COT group (n = 101)	HFNCO group (n = 102)	*t*/χ^2^ value	*P* -value
Age (yr)	56.51 (54.49, 58.42)	57.75 (56.08, 59.44)	−0.946	.345
Sex			0.04	.842
Male	62 (62.7)	64 (63.3)		
Female	39 (38.3)	38 (38.7)		
Smoking history			0.04	.842
Yes	32 (31.3)	31 (31.7)		
No	69 (69.7)	71 (70.3)		
Comorbidities (HTN/DM/HLP)	46 (50.7)	56 (51.3)	1.777	.182
Operative duration (h)	5.6 (5.4, 5.79)	5.84 (5.57, 6.1)	−1.464	.145
Mechanical ventilation (d)	4.36 (4.07, 4.62)	4.13 (3.82, 4.46)	1.061	.229
APACHE-II score	15.47 (14.68, 16.24)	15.19 (14.57, 15.83)	0.54	.279
Pre-extubation (0.5 h)				
HR (beats/min)	97.21 (94.4, 100.04)	97.48 (94.76, 100.41)	−0.132	.895
RR (breaths/min)	22.12 (21.4, 22.82)	22.37 (21.25, 24.06)	−0.318	.752
MAP (mm Hg)	84.02 (82.12, 86.03)	84.16 (82.091, 86.29)	−0.96	.924
SpO_2_ (%)	97.49 (97.25, 97.72)	97.13 (96.84, 97.4)	1.895	.06
pH	7.43 (7.42, 7.45)	7.41 (7.4, 7.42)	2.691	<.001
PaO_2_ (mm Hg)	114.35 (108.73, 120.37)	109.75 (104.9, 114.93)	1.153	.251
PaCO_2_ (mm Hg)	37.3 (35.97, 38.72)	37.2 (36.24, 38.32)	0.123	.902
P/F ratio	229.02 (219.6, 237.69)	237.52 (223.68, 254.05)	−0.942	.348

Categorical data is displayed as O(E), Non-normal distribution data is displayed as median (Q1, Q3).

APACHE-II = Acute Physiology and Chronic Health Evaluation II, COT = conventional oxygen therapy, DM = diabetes mellitus, HFNCO = high-flow nasal cannula oxygenation, HLP = hyperlipidemia, HR = heart rate, HTN = hypertension, MAP = mean arterial pressure, P/F = oxygenation index, PaCO_2_ = partial pressure of carbon dioxide in arterial blood, PaO_2_ = partial pressure of oxygen, pH = potential of hydrogen, RR = respiratory rate, SpO_2_ = pulse oxygen saturation.

### 3.2. Comparison of HR, MAP, and RR at 6, 12, and 24 hours post-extubation

No significant differences were observed in HR or MAP between the COT and HFNCO groups at 6, 12, or 24 hours post-extubation (*P* > .05). However, the HFNCO group exhibited significantly lower RR than the COT group at 6 hours (*P* < .05), though this difference diminished at 12 and 24 hours (*P* > .05). Data are summarized in Table 2.

**Table 2:** **Post-extubation HR, RR, and MAP comparisons between groups M (Q1, Q3**).

Variable	Time post-extubation (h)	COT group (n = 101)	HFNCO group (n = 102)	*t* value	*P* -value
HR (beats/min)	6	102.04 (98.56, 105.88)	98.21 (94.61, 102.49)	−1.431	.155
	12	99.81 (96.21, 103.33)	96.94 (93.12, 100.98)	−1.055	.293
	24	98.62 (95.17, 102.25)	95.06 (91.3, 99.31)	−1.253	.212
RR (breaths/min)	6	22.19 (21.45, 22.89)	20.16 (19.51, 20.82)	4.099	<.001
	12	20.24 (19.19, 20.91)	19.72 (19.19, 20.29)	1.169	.244
	24	19.9 (19.31, 20.57)	19.82 (19.26, 20.44)	0.177	.86
MAP (mm Hg)	6	87.76 (85.09, 90.47)	86.56 (84.19, 88.75)	−0.610	.542
	12	85.37 (82.92, 87.52)	84.1 (82.04, 86.1)	−0.764	.446
	24	88.77 (86.58, 91.16)	86.14 (83.51, 88.88)	−1.458	.147

Non-normal distribution data is displayed as median (Q1, Q3).

HR = heart rate, MAP = mean arterial pressure, RR = respiratory rate.

### 3.3. Blood gas analysis at 6, 12, and 24 hours post-extubation

No significant differences were observed in SpO_2_ or PaO_2_ between groups at any time point (*P* > .05). However, the HFNCO group demonstrated higher pH values at 12 and 24 hours (*P* < .05) and lower PaCO_2_ levels at all time points (*P* < .05). Additionally, the P/F ratio was significantly higher in the HFNCO group compared to the COT group (*P* < .05). Results are detailed in Table 3.

**Table 3:** **Post-extubation blood gas comparisons between groups M (Q1, Q3**).

Variable	Time post-extubation (h)	COT group (n = 101)	HFNCO group (n = 102)	*t* value	*P*-value
pH	6	7.46 (7.44, 7.47)	7.42 (7.41, 7.43)	3.523	<.001
	12	7.42 (7.41, 7.43)	7.45 (7.44, 7.46)	−3.351	<.001
	24	7.43 (7.42, 7.44)	7.45 (7.44, 7.45)	−2.831	.005
SpO_2_ (%)	6	96.42 (95.97, 96.84)	97.15 (96.75, 97.57)	2.507	.013
	12	96.73 (96.12, 97.27)	97.09 (96.73, 97.43)	0.939	.349
	24	96.45 (95.55, 97.14)	96.64 (95.62, 97.36)	0.304	.762
PaO_2_ (mm Hg)	6	104.16 (96.76, 112.46)	112.29 (103.30, 120.83)	1.388	.167
	12	109.43 (103.41, 116.49)	108.38 (100.95, 115.35)	−0.212	.832
	24	114.15 (101.21, 132.11)	105.98 (99.31, 113.17)	−0.958	.340
PaCO_2_ (mm Hg)	6	40.35 (38.62, 42.13)	36.66 (35.48, 37.86)	3.401	<.001
	12	40.13 (38.75, 41.51)	37.22 (36.14, 38.48)	3.086	.002
	24	39.64 (38.21, 40.99)	36.95 (35.9, 38.02)	3.073	.003
P/F ratio (mm Hg)	6	237.45 (219.82, 256.13)	298.05 (272.59, 325.17)	3.949	<.001
	12	235.92 (220.06, 254.38)	298.32 (277.84, 318.48)	4.574	<.001
	24	238.46 (220.79, 257.89)	287.16 (262.82, 314.46)	3.137	.002

Non-normal distribution data is displayed as median (Q1, Q3).

P/F = oxygenation index, PaCO_2_ = partial pressure of carbon dioxide in arterial blood, PaO_2_ = partial pressure of oxygen, pH = potential of hydrogen, SpO_2_ = pulse oxygen saturation.

### 3.4. Clinical outcomes: reintubation, hospital stay, ICU delirium, and mortality

Compared to the COT group, the HFNCO group was associated with significantly lower rates of reintubation, shorter total hospital stays, reduced ICU stays, and lower ICU delirium incidence (*P* < .05). However, no significant difference was observed in 28-day mortality between groups (*P* > .05). Data are presented in Table 4.

**Table 4:** **Clinical outcomes between groups O(E)/M (Q1, Q3**).

Group	Reintubation rate	Total hospital stay (d)	ICU stay (d)	28-d mortality	CAM-ICU incidence
COT	14 (8)	33.07 (28.38, 39.47)	7.32 (6.22, 8.73)	2 (1)	17 (11.1)
HFNCO	2 (8)	23.69 (22.06, 25.47)	3.21 (2.7, 4)	0 (1)	5 (10.9)
*t*/χ^2^	9.642	−3.176	−5.441	2.773	7.209
*P*-value	.002	.002	<.001	.096	.007

Categorical data is displayed as O(E), non-normal distribution data is displayed as median (Q1, Q3).

CAM-ICU = confusion assessment method for the intensive care unit, COT = conventional oxygen therapy, HFNCO = high-flow nasal cannula oxygenation.

## 4. Discussion

Postoperative hypoxemia following cardiac surgery, frequently attributed to pulmonary edema, atelectasis, or infection,^[7]^ remains a critical challenge requiring prompt and effective respiratory support. This study found that HFNCO was associated with better oxygenation, reduced respiratory effort, and shorter recovery time compared to COT. The observed reduction in RR at 6 hours post-extubation (*P* < .05) aligns with HFNCO’s ability to mitigate inspiratory resistance and provide low-level positive end-expiratory pressure (1–8 cm H_2_O), thereby enhancing alveolar recruitment and gas exchange.^[8]^ These findings corroborate recent meta-analyses highlighting HFNCO’s role in reducing work of breathing and improving patient comfort in postoperative settings.^[9]^

The superior oxygenation indices (P/F ratio: 298.05 vs 237.45 at 6 hours, *P* < .001) and lower PaCO_2_ levels (36.66 vs 40.35 mm Hg, *P* < .001) in the HFNCO group are consistent with its known physiological advantages, including dead space reduction and enhanced mucociliary clearance.^[10]^ These mechanisms are particularly relevant in cardiac surgery patients, where postoperative pulmonary complications are exacerbated by impaired cough reflex and pain-induced shallow breathing.^[11]^ Recent randomized trials have similarly reported that HFNCO reduces the incidence of atelectasis and pneumonia in this population, further validating our results.^[12]^

Clinically, the use of HFNCO was associated with a markedly lower reintubation rate (2% vs 14%, *P* = .002) and shorter ICU stays (3.21 vs 7.32 days, *P* < .001), outcomes consistent with Hernández et al’s landmark trial.^[13]^ The reduction in ICU delirium (5% vs 17%, *P* = .007) may be attributed to HFNCO’s ability to stabilize sleep patterns by minimizing mask-related discomfort and noise. However, the lack of difference in 28-day mortality (*P* = .096) aligns with emerging evidence that HFNCO’s benefits are primarily confined to short-term physiological and resource utilization outcomes.^[14]^

Despite these advantages, HFNCO is not without risks. Delayed escalation to invasive mechanical ventilation in HFNCO-treated patients has been associated with adverse outcomes in hypoxemic respiratory failure.^[15]^ A 2023 multicenter cohort study reported that prolonged HFNCO use (>48 hours) without clinical improvement increased mortality by 18% in postoperative cardiac patients.^[16]^ This underscores the necessity for vigilant monitoring and predefined escalation protocols, such as the ROX index (respiratory rate-oxygenation), to guide timely transitions to invasive support.^[17]^

The European Society of Intensive Care Medicine (ESICM) recently endorsed HFNCO as first-line therapy for post-cardiac surgery hypoxemia, emphasizing its role in reducing ICU resource strain.^[18,19]^ However, our study’s retrospective design and single-center cohort limit generalizability. Future research should prioritize prospective, multicenter randomized controlled trials to validate these findings and refine HFNCO parameters (e.g., optimal flow rates, FiO_2_ titration).^[20]^ Additionally, cost-effectiveness analyses are needed to evaluate HFNCO’s economic impact, particularly in resource-limited settings.^[21]^

## 5. Conclusion

High-flow nasal cannula oxygen therapy may represent a paradigm shift in postoperative respiratory management, as its use was associated with tangible benefits in oxygenation, patient comfort, and ICU efficiency. Its integration into standardized postoperative care pathways, coupled with rigorous monitoring protocols, could further optimize outcomes. Nevertheless, clinicians must balance its advantages against the risk of delayed intervention, ensuring individualized patient care aligned with evolving evidence. Limitations include lack of retrospective collection of inflammatory biomarkers; future studies should incorporate serial CRP and procalcitonin measurements to correlate physiological improvements with systemic inflammation.

## Author contributions

**Conceptualization:** Kaifeng Luo.

**Data curation:** Kaifeng Luo.

**Formal analysis:** Kaifeng Luo.

**Funding acquisition:** Kaifeng Luo.

**Investigation:** Kaifeng Luo, Gang Li.

**Methodology:** Kaifeng Luo.

**Project administration:** Chengli Wang.

**Resources:** Chengli Wang.

**Software:** Chengli Wang.

**Supervision:** Gang Li, Chengli Wang.

**Writing – original draft:** Kaifeng Luo.

**Writing – review & editing:** Kaifeng Luo.
